# Anticancer actions of lysosomally targeted inhibitor, LCL521, of acid ceramidase

**DOI:** 10.1371/journal.pone.0177805

**Published:** 2017-06-14

**Authors:** Aiping Bai, Cungui Mao, Russell W. Jenkins, Zdzislaw M. Szulc, Alicja Bielawska, Yusuf A. Hannun

**Affiliations:** 1 Department of Biochemistry & Molecular Biology, Medical University of South Carolina, Charleston, South Carolina, United States of America; 2 Department of Medicine, Stony Brook University, Stony Brook, New York, United States of America; 3 Stony Brook Cancer Center, Stony Brook, New York, United States of America; Universite Paris Diderot-Paris7 - Batiment des Grands Moulins, FRANCE

## Abstract

Acid ceramidase, which catalyzes ceramide hydrolysis to sphingosine and free fatty acid mainly in the lysosome, is being recognized as a potential therapeutic target for cancer. B13 is an effective and selective acid ceramidase inhibitor *in vitro*, but not as effective in cells due to poor access to the lysosomal compartment. In order to achieve targeting of B13 to the lysosome, we designed lysosomotropic N, N-dimethyl glycine (DMG)-conjugated B13 prodrug LCL521 (1,3-di-DMG-B13). Our previous results indicated the efficient delivery of B13 to the lysosome resulted in augmented effects of LCL521 on cellular acid ceramidase as evaluated by effects on substrate/product levels. Our current studies indicate that functionally, this translated into enhanced inhibition of cell proliferation. Moreover, there were greater synergistic effects of LCL521 with either ionizing radiation or Tamoxifen. Taken together, these results clearly indicate that compartmental targeting for the inhibition of acid ceramidase is an efficient and valuable therapeutic strategy.

## Introduction

Acid ceramidase (ACDase), a lysosomal enzyme, catalyzes the hydrolysis of ceramide (Cer) to sphingosine (Sph) and free fatty acid, and Sph can then ‘escape’ the lysosome to be re-acylated to Cer or to be utilized by Sph kinases to form sphingosine-1-phosphate (S1P). Cer, Sph and S1P, three of the major sphingolipid metabolites, are involved in regulation of cell survival, proliferation and death, in addition to many other cell responses [1–3]. Cer is known to be a key modulator of cancer cell growth and apoptosis. Sph has functional roles in regulating the actin cytoskeleton, endocytosis, cell cycle and apoptosis. Conversely, S1P acts as an anti-apoptotic tumor protective agent. Therefore, ACDase emerges as an important enzyme because of its essential role in regulating Cer-Sph-S1P inter-metabolism. Inhibition of ACDase increases ceramide and decreases Sph and possibly S1P [4–6]. Furthermore, ACDase overexpression could provoke tumor cell resistance to chemotherapy and radiation therapy [7,8]. Thus, inhibition of ACDase sensitizes tumors to chemo and radiotherapy, and emerges as a very promising strategy for cancer therapeutics.

Our group has been focusing on the development of ACDase inhibitors for many years. Based on substrate similarity, a diverse set of compounds established on the structural skeleton of phenyl amino alcohol were designed and validated [9–13]. Among them, B13, (1R, 2R)-1-(4’-nitrophenyl)-2-(tetradecanoyl-amido)-1, 3-propandiol, was identified as a potent ACDase inhibitor *in vitro*. The selective inhibition of ACDase renders B13 a potential leading molecule to evaluate ACDase biology and therapeutics [14–16]. However, B13’s powerful inhibitory effect may not be fully obtained in cells since the neutral and lipophilic chemical characteristics may compromise B13’s ability to enter the lysosomal compartment where ACDase predominantly resides. To improve the solubility and compartmental targeting of B13, we first attempted to perform some structural modifications; however, these slight structural changes (such as LCL204 and LCL 385) mostly resulted in a decrease in the ability of B13 to inhibit ACDase. Therefore, we pursued a distinct strategy in designing Di-DMG B13 prodrug, LCL521, and our previous results indicated that LCL521 could enhance cellular uptake by rendering the molecule more water-soluble and by enhancing lysosomal sequestration *via* manipulating the polarity of the prodrug and its ionization [17].

In this study, we utilize MCF7 human breast adenocarcinoma cell to evaluate the cellular action of LCL521. The implications of these results are discussed.

## Materials and methods

### Chemicals

All solvents and general reagents were purchased from Sigma-Aldrich and Fluka. B13 and LCL521 were synthesized in the Lipidomic Core, Medical University of South Carolina [17, 18]. Mass spectral data were recorded in a positive ion electrospray ionization (ESI) mode on Thermo Finnigan TSQ 7000 triple quadrupole mass spectrometer, and processed as previous described [19].

### Cell culture

#### Cell lines

MCF7 cells were purchased from American Type Culture Collection (ATCC, Rochville, MD) and were cultured in RPMI 1640 (Life Technologies, Inc.), supplemented with 10% fetal bovine serum, 100 unit/ml penicillin and 100 ug/ml streptomycin, and were grown in a humidified incubator with 5% CO_2_ at 37°C. Tamoxifen resistant MCF7 cells were generated by treating wild MCF7 with gradually rising dose of Tamoxifen, and were supplemented with 10% Charcoal stripped fetal bovine serum. Serum was made according to reference with little modification (C6241, Sigma-aldrich). Briefly, 50ml of fetal bovine serum was treated with 1g dextran-coated charcoal at 4°C for 12h with occasionally agitating, then centrifuge at 500g for 10min, the obtained supernatant was centrifuge at 1000g for 10min and repeated one more time before filtrated through 0.45μm and 0.22 μm of pore size filter. Obtained serum was stored at -20°C. MTT assay was applied once per month to confirm tamoxifen dose (around IC90) before move to the closest higher dose.

#### Clonogenic assay

MCF7 cells 300/dish were cultured in 35mm dish overnight before single dose of 2.0Gy ionizing radiation (IR). 1h after IR, testing compound was added. For the 5-time treatment, every 24h, media was replaced with fresh media together with same amount of testing compound. After then, cells were kept cultured for total of 4 weeks before stained with crystal violet (1g/500ml formalin). Colonies was counted by Clono-Counter (http://java.sun.com) with threshold 160 and gray width 15.

#### Cell cycle analysis

MCF7 cell 1x10^5^/well/6well plate was cultured overnight before treatment with the various doses of inhibitors. After 24h, cells were harvested and washed with cold PBS before adding 5ml 70% ethanol. Samples were kept at 4°C overnight, and then they were centrifuged at 850g for 5 min and washed with cold PBS 2 times. 500μl RNase (R-5125, Sigma, 2mg/ml) and PI solution (P-4170, Sigma, 0.1mg/ml in 0.6% Triton X-100) were added and kept in the dark for another 45min before the FACS analysis.

#### Cell viability assay

The MTT assay [11] was used to quantify viable cells.

## Results and discussion

### LCL521 represents an acute and potent inhibitor of ACDase

**Fig 1:**
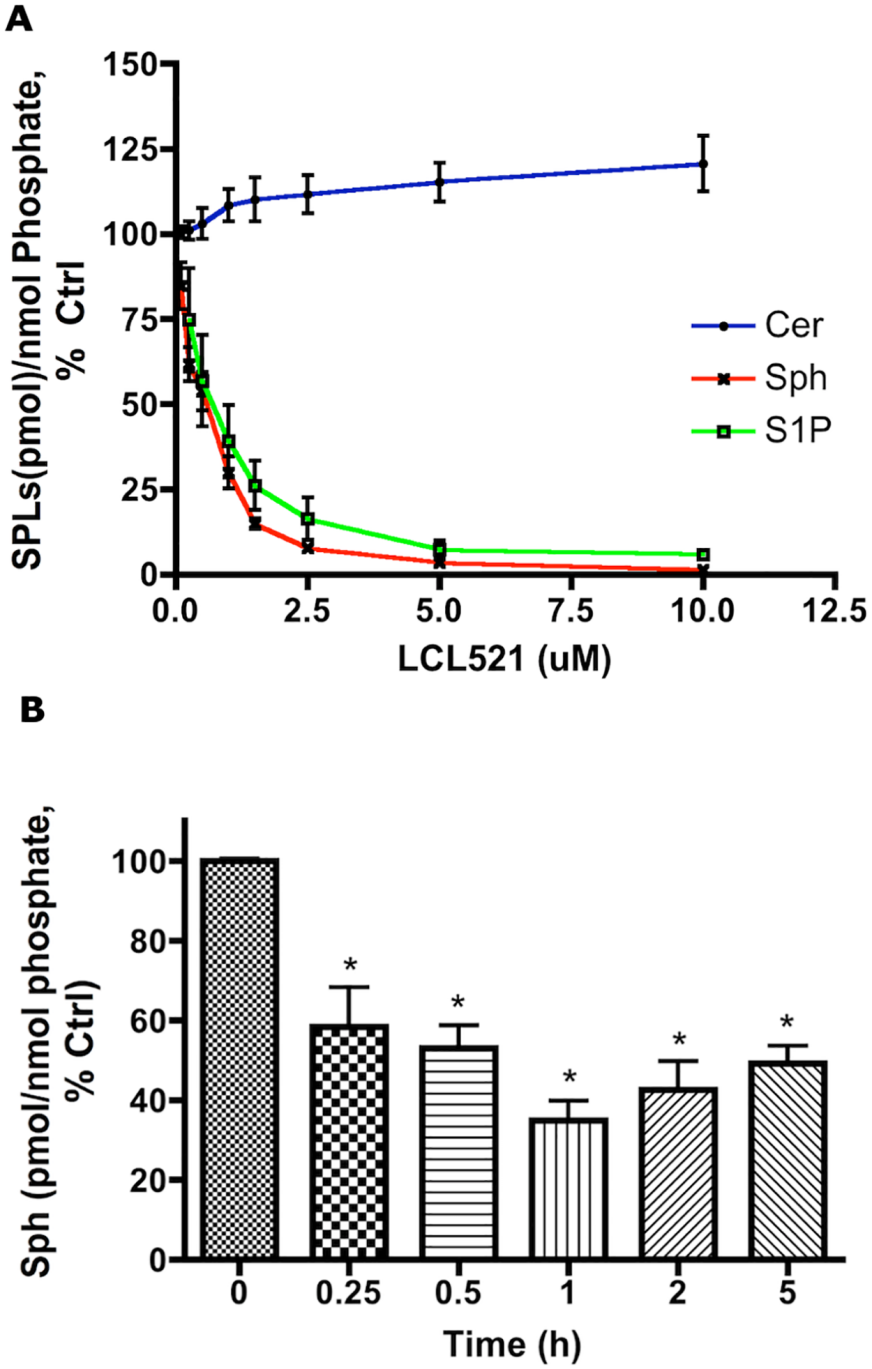
LCL521 represents an acute and potent inhibitor of ACDase. **(A)** MCF7 cells were treated with vehicle, or with 0.1, 0.25, 0.5, 1, 1.5, 2.5, 5, and 10μM LCL521 for 1h. Cer, Sph and S1P were then extracted and quantified by LC-MS/MS. (n = 4, three times experiments with one time duplicates and two times single experiment). The actual amounts of Cer, Sph and S1P after treatment with LCL521 are presented in S1B–S1D Fig (**B**). MCF7 cells were treated with vehicle or 1μM LCL521 for 15min, 30min, 1, 2, and 5h. Sph was then extracted and quantified by LC-MS/MS. (*p<0.05, n = 7, 3 times experiments with 2 times duplicates and 1 time triplicates).

Our previous results indicated that B13 specifically inhibits ACDase *in vitro* but the inhibitory effect is not fully realized in cells. The effects of B13 on cellular sphingolipids were modest in MCF7 breast adenocarcinoma cells, demonstrating that after 1h incubation, the decrease of Sph was hardly achieved even at the concentration of 30μM [S1A Fig]. Because B13 is an aromatic ceramide analog, inefficiency in compartmental targeting would augment the possibilities of additional targets, especially enzymes related to ceramide metabolic pathway. In contra distinction, a decrease of endogenous Sph as well as S1P were observed starting at 100nM LCL521, the lowest concentration tested, with more profound effects at 1–5 μM, where we also observed increase of endogenous Cer, all after 1 h of treatment [Fig 1A]. In a time course study, 1μM LCL521 diminished endogenous Sph by over 66% after only 15min incubation [Fig 1B]. Since Sph is only generated from Cer by ceramidase [20], these results demonstrate LCL521 an acute and potent inhibitor of ACDase.

### LCL521 in combination with single dose of IR demonstrates synergistic effects

**Fig 2:**
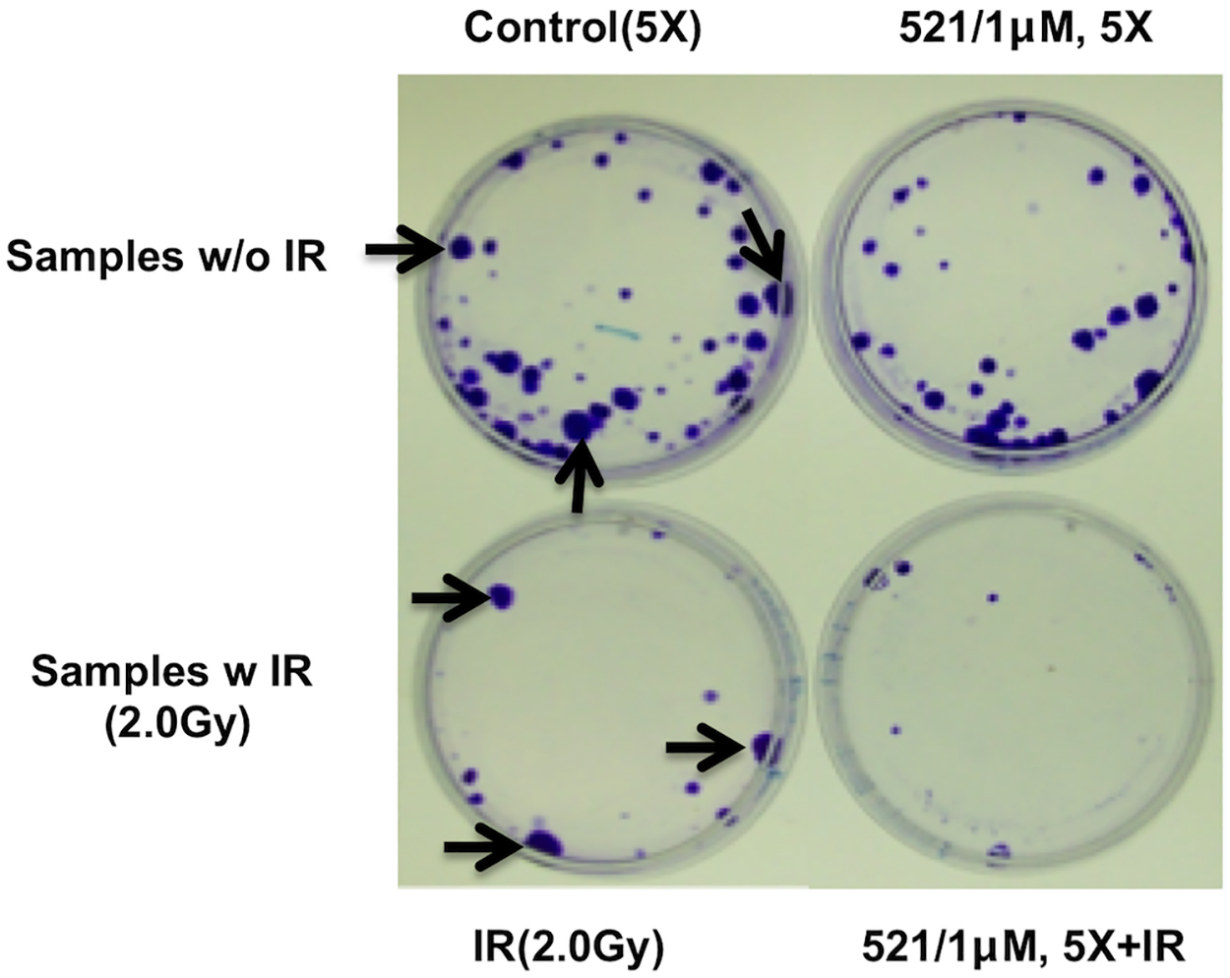
Synergistic effect of LCL521 and single dose of ionizing radiation. MCF7 cells were irradiated with 0 or 2Gy using 137 Cesium irradiator. 1h after IR, each replicated treatment were further treated with vehicle or 5x 1μM LCL521. For the 5-time treatment, media were replaced every 24h with fresh media that contained either vehicle or 1μM LCL521. After that, cells were cultured for 4 weeks and then stained with crystal violet (1g/500ml formalin). Representative crystal staining is shown. Colonies count is presented in S2 Fig.

To define biologic consequences of inhibition of ACDase, the effects of a combination of LCL521 and single low dose of ionizing radiation (IR) were evaluated through a long-term clonogenic assay. IR was chosen as it has been clearly shown to involve ACDase [7]. Clonogenic assays have proven to be particularly helpful in determining the effects of radiation on the tumor cells [21]. In our studies, after long-term culture (4 weeks after the treatment), single dose of IR showed strong effects on MCF7 cells killing as revealed by the large decrease in the number of colonies after IR. However, these doses of IR showed no effects on the proliferation of the surviving cells because the size of formed colonies was the same with or without IR [black arrow, Fig 2]. When combined with the 5-time 1μM LCL521 treatment, we observed a decrease in both colony formation and size, the latter suggesting an effect on cell proliferation.

### LCL521 induces G1 cell cycle arrest and improves B13’s cytotoxicity on MCF7 cells

**Fig 3:**
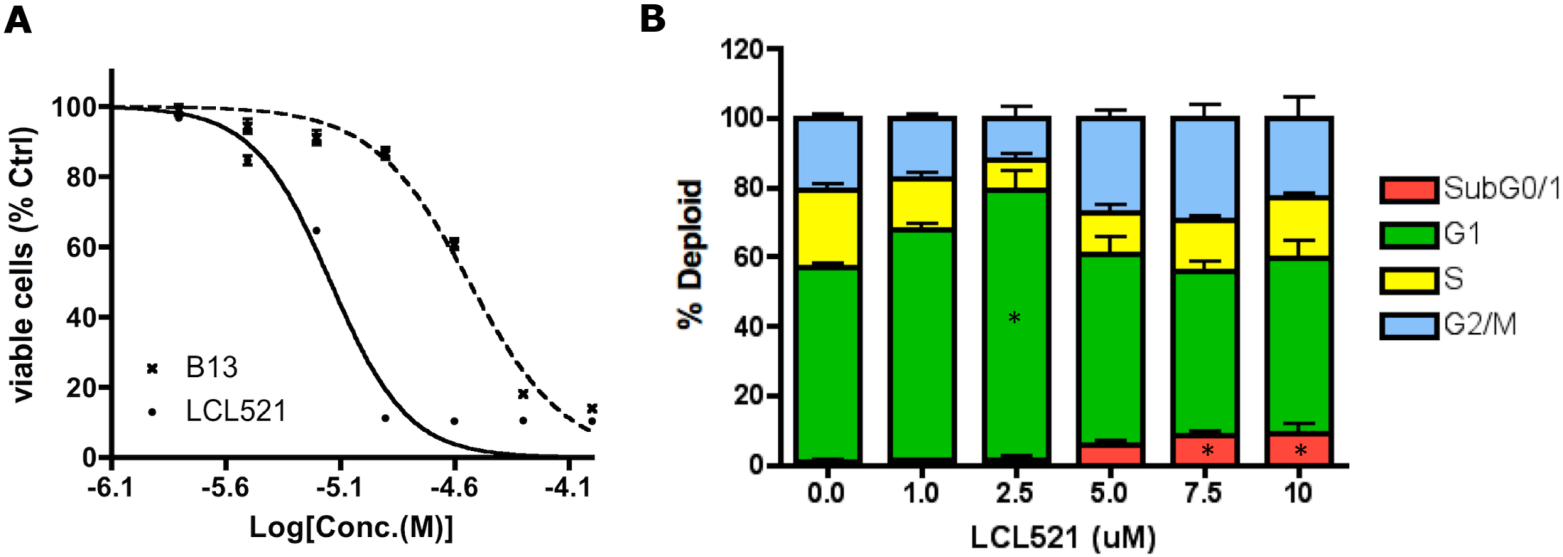
LCL521 demonstrates significant effects on MCF7 cells cytotoxicity and proliferation. (**A**) **Viable cell analysis**. MCF7 cells were treated with vehicles, 0.78, 1.56, 3.125, 6.25,12.5, 25, 50, and 100μM of either B13 or LCL521 for 48h and then MTT assays were performed. The results are expressed as a percentage relative to untreated cells and are presented as means ± st dev. of single experiment with 4 time replicates. (**B**) **Effect of LCL521 on MCF7 cell cycle**. MCF7 cells were treated with vehicle or with 1, 2.5, 5, 7.5 and 10 μM LCL521 for 24h. Cells were fixed with 70% ethanol overnight before adding 500μL RNase and PI solution. Samples were kept in the dark for another 45min before the FACS analysis. (n = 4, two times experiments with duplicates in each. * *p*<0.01). Representative flow cytometric analyses are shown in S3 Fig.

Based on the above results, it was necessary to evaluate the effects of LCL521 on MCF7 cell proliferation. LCL521 inhibited the growth of MCF7 cells in a dose dependent manner [Fig 3A & Table 1]. Results demonstrated that LCL521 highly improved upon B13’s cytotoxicity on MCF7 cells. Next, the effects of LCL521 on cell cycle analysis progression were studied. Results demonstrated that after only 24h incubation, low dose of LCL521 clearly induced G1 cell cycle arrest with a marked decrease of cells in S phase. When the concentration reached higher dose range (>5μM), subG0/1 is elevated, which is clearly induction of apoptosis. Thus, LCL521 induces on its own a strong effect on cell cycle [Fig 3B]. These results demonstrate significant effects of LCL521 on cell proliferation and cell cycle progression.

**Table 1:** IC_50_ values for B13 and LCL521.

IC50 (uM)	24h	48h	72h
B13	40.64±1.031	28.97±1.036	24.66±1.019
LCL521	11.91±1.094	7.18±1.042	7.46±1.033

MCF7 cells were treated with vehicles, 0.78, 1.56, 3.125, 6.25,12.5, 25, 50, and 100μM of either B13 or LCL521 for 24, 48 and 72h and then MTT assays were performed. The results are expressed as a percentage relative to untreated cells and are presented as means ± st dev. of single experiment with 4 time replicates.

### LCL521 sensitizes Tamoxifen resistant MCF7 cells to Tamoxifen treatment

**Fig 4:**
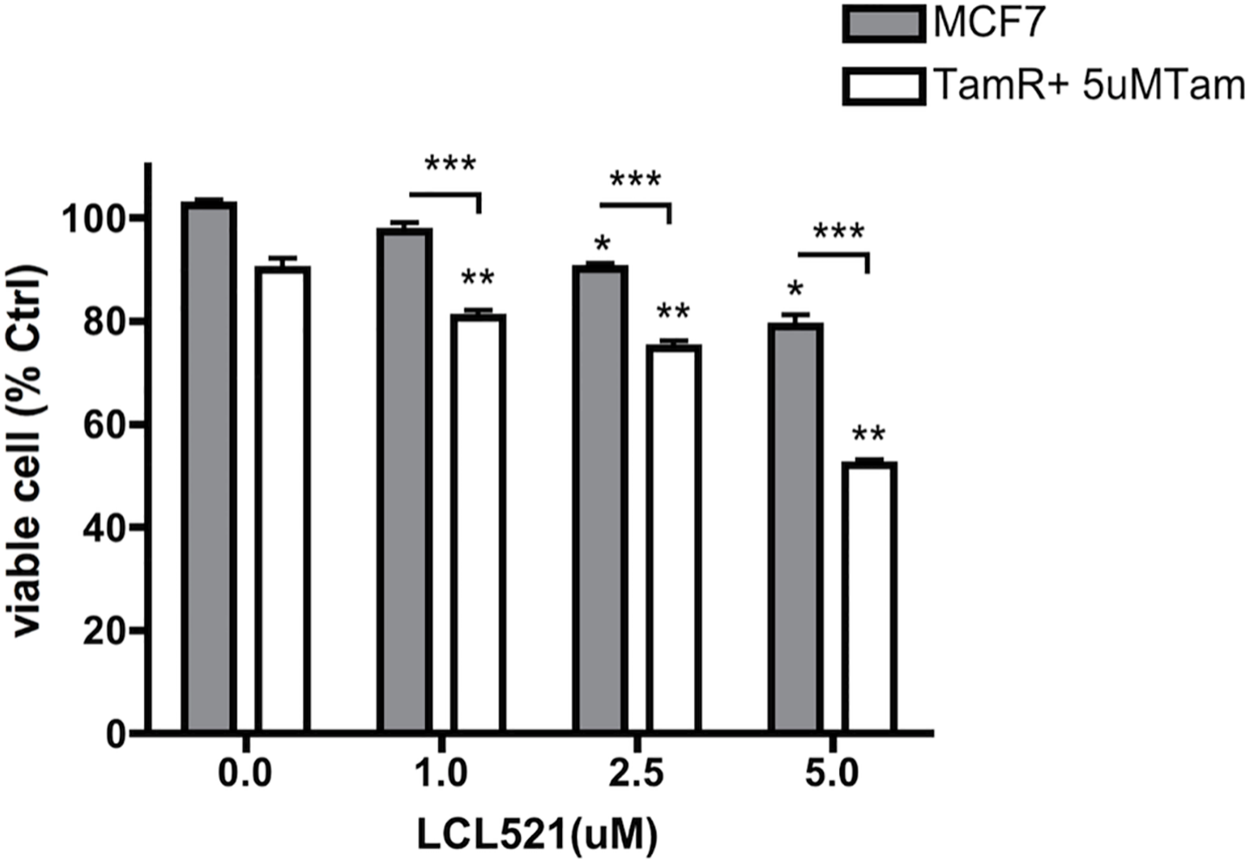
Synergistic effects of LCL521 and Tamoxifen on Tamoxifen resistant MCF7 cells. Tamoxifen resistant MCF7 cells (TamR) were plated in 96 well plate overnight before treatment with 10μM Tamoxifen (final concentration is 5μM) for 1h, then treated with LCL521 with the final concentration at 0, 1, 2.5 and 5μM. To compare LCL521 its own killing effect, wild MCF7 cells were plated in 96 well plate at same time and varies dose of LCL521 were treated at the same time. Cells were kept for 48h before MTT assay was preformed. The results are expressed as a percentage of vehicle samples and presented as means ± st dev. of triplicates. * *p*<0.05 (vs 0); ** *p*<0.01 (vs 0); *** *p*<0.001 (TamR vs MCF7).

Finally, we evaluated the combination of LCL521 and Tamoxifen, an antagonist of the estrogen receptor (ER) used in ER positive breast cancer [22]. Primary or acquired resistance to Tamoxifen remains a major challenge in the treatment of ER positive breast cancer. Therefore, we investigated whether LCL521 could sensitize Tamoxifen resistant cells to its treatment. For this purpose, we first generated Tamoxifen resistant MCF7 cell line (TamR) by treating wild MCF7 with low dose of Tamoxifen, which was increased gradually over time (0.5–10μM) for a total of 8 months. Then, we utilized this resistant cell line for the combination experiment. After 48h incubation, the combination of LCL521 and Tamoxifen produced much stronger cell killing when compared with the control, compared to a small effect of LCL521 on its own and a modest inhibitory effect of 5μM Tamoxifen (10.1%) [Fig 4].

## Conclusions

The synthesis of B13, also named D-*NMAPPD*, was first published in1992 [18], and later was found to inhibit ACDase [15]. Our previous data show that B13 is not only an effective but also a specific inhibitor of ACDase *in vitro*, with no activity against neutral or alkaline CDases [17]. On the other hand, B13 shows modest effects on ACDase in cells, which led us to synthesize prodrugs of B13 that target it to the lysosome. Importantly, the results in the study show that the Di-DMG B13 prodrug LCL521 is very active in cells against ACDase, and it displays biological anti-cancer activities.

The first major conclusion from this study relates to the success of improving the effectiveness of B13 through compartmental targeting. It is increasingly appreciated that sphingolipid signaling is highly compartmentalized such those actions of various regulated enzymes of sphingolipids metabolism, including sphingomyelinases and ceramidases, are governed by their compartment of action [23]. In order to probe these distinct pathways, it becomes necessary to establish chemical probes that target these pathways in their relevant subcellular compartments. Therefore, we desired to improve on the inhibition of lysosomal ACDase that is known to be important in regulation of survival of cancer cells and their responses to chemo- and ionizing therapy. Our previous studies showed that although B13 is quite active and specific in inhibiting ACDase *in vitro*, this was not translated to effectiveness in cells, presumably because of poor access to the lysosome, the main compartment for action of ACDase. Based on that, we set out to improve the action of B13 by developing a chemical approach that allows delivery of a prodrug to the lysosome and liberation of B13. Thus, Di-DMG B13 prodrug was designed with ionizable amines (in the DMG moiety) that result in lysosomal concentration [17]. In turn, the DMG groups are easily de-esterified, liberating a free B13. Indeed, our previous metabolic and cell studies revealed significant uptake of the LCL521, and significant compartmental accumulation of B13 as the ‘liberated’ drug [17]. Importantly, the results show that LCL521 exhibits significant activity on cellular ACDase, resulting in marked depletion of endogenous Sph. These metabolic effects coincided with the liberation of the free B13. Functionally, LCL521 exhibited significant effects on G1 cell cycle arrest, and cell death. All these results validate the rationale of our approach in developing cellular active B13 prodrug.

In an independent approach, we had previously designed and synthesized pyridinium ceramides that target ceramide analogs to the mitochondria, thus allowing the probing of ceramide functions in mitochondria [24]. On the other hand, the functions of ceramide at the plasma membrane can be probed through the application of extracellular sphingomyelinases and ceramidases [25]. Thus, taken together, there are now increasing tools to probe the action of bioactive sphingolipids in various compartments.

Another important conclusion from this study pertains to the effectiveness of inhibition of ACDase in tumor cell killing. A previous study showed that radiation-resistant glioblastoma cell line U87 (LXSN, -M E6) exhibited elevated levels of ACDase expression when exposed to γ-radiation, and inhibition of ACDase sensitized these cells to γ-radiation and increased apoptosis [26]. Down-regulation of ACDase using siRNA sensitized head and neck cancer cells to Fas-induced apoptosis whereas overexpression of ACDase increased the resistance to Fas-induced cell killing [27]. The Inhibition of ACDase in three human hepatoma cell lines in conjunction with vinblastine or doxorubicin resulted in significantly decreased cell survival compared to treatment with vinblastine or doxorubicin alone, and this sensitization to chemotherapeutic drugs was replicated *in vivo* using ACDase siRNA [28]. Addition of an ACDase inhibitor (LCL521) to mouse model of xenograft irradiation produced radio-sensitization and prevention of tumor relapse [7]. Recent studies also indicate that LCL521 targets lysosomes to activate cathepsin B and cathepsin D, resulting in interrupted autophagy and ER stress that culminates in myeloid derived suppressor cells death [29]. These results then suggest that ACDase could be an important target in cancer therapy, and thus small molecule specific inhibitors of ACDase are needed. In this context, the current results show that LCL521 on its own resulted in cell cycle arrest. In combination with tamoxifen or ionizing radiation, LCL521 exhibited significant additive effects on tumor proliferation and death.

Taken together, the results show that the cellular and biological effects of B13 can be augmented through its prodrug LCL521 that target it to the lysosome. Thus, LCL521 significantly Inhibited ACDase, which sensitized tumor cells to chemo- and radiotherapy. This then promises to be a useful strategy for cancer therapeutics.

## Statistical analysis

Where indicated, data were represented as mean ± SD. Statistical analysis was performed using Graphpad ANOVA, with *p*-value <0.05 considered statistically significant.

## Supporting information

S1 Fig LCL521 represents an acute and potent inhibitor of ACDase. (**A**) MCF7 cells were treated with vehicle, or with 0.1, 0.25, 0.5, 1, 2.5, 5 and 10μM LCL521 for 1h, or with 1, 5, 10, 20, 30 μM B13 for 1h. Sph were then extracted and quantified by LC-MS/MS. (n = 2, two times experiments); (**B**). LCL521’s dose response on endogenous Cer. (1h, n = 2 two times experiments); (**C**). LCL521’s dose response on endogenous Sph. (1h, n = 2, two times experiments, * *p*<0.05, *vs* Ctrl); (**D**). LCL521’s dose response on endogenous S1P. (1h, n = 2, two times experiments, * *p*<0.05, *vs* Ctrl).
(TIFF)

S2 Fig Synergistic effect of LCL521 and single dose of ionizing radiation. MCF7 cells were irradiated with 0 or 2Gy using 137 Cesium irradiator. 1h after IR, each replicated treatment were further treated with vehicle or 5x1μM LCL521. For the 5-time treatment, media were replaced every 24h with fresh media that contained either vehicle or 1uM LCL521. After that, cells were cultured for 4 weeks and then stained with crystal violet (1g/500ml formalin). (n = 2, two times experiments, * *p*<0.01, ** *p*<0.001).
(TIFF)

S3 Fig LCL521’s effect on MCF7 cell cycle. Cells were treated with vehicle or 1, 2.5, 5, 7.5 and 10μM LCL521 for 24h. Cells were then fixed with 70% ethanol overnight before adding 500μL RNase and PI solution. Samples were kept in the dark for another 45min before the FACS analysis. Representative flow cytometric analyses are shown.
(TIFF)
