# Supplementary figures and images for: Anticancer actions of lysosomally targeted inhibitor, LCL521, of acid ceramidase
Source: PLoS One. 2017 Jun 14;12(6):e0177805. doi: 10.1371/journal.pone.0177805 (PMC5470663; doi:10.1371/journal.pone.0177805)

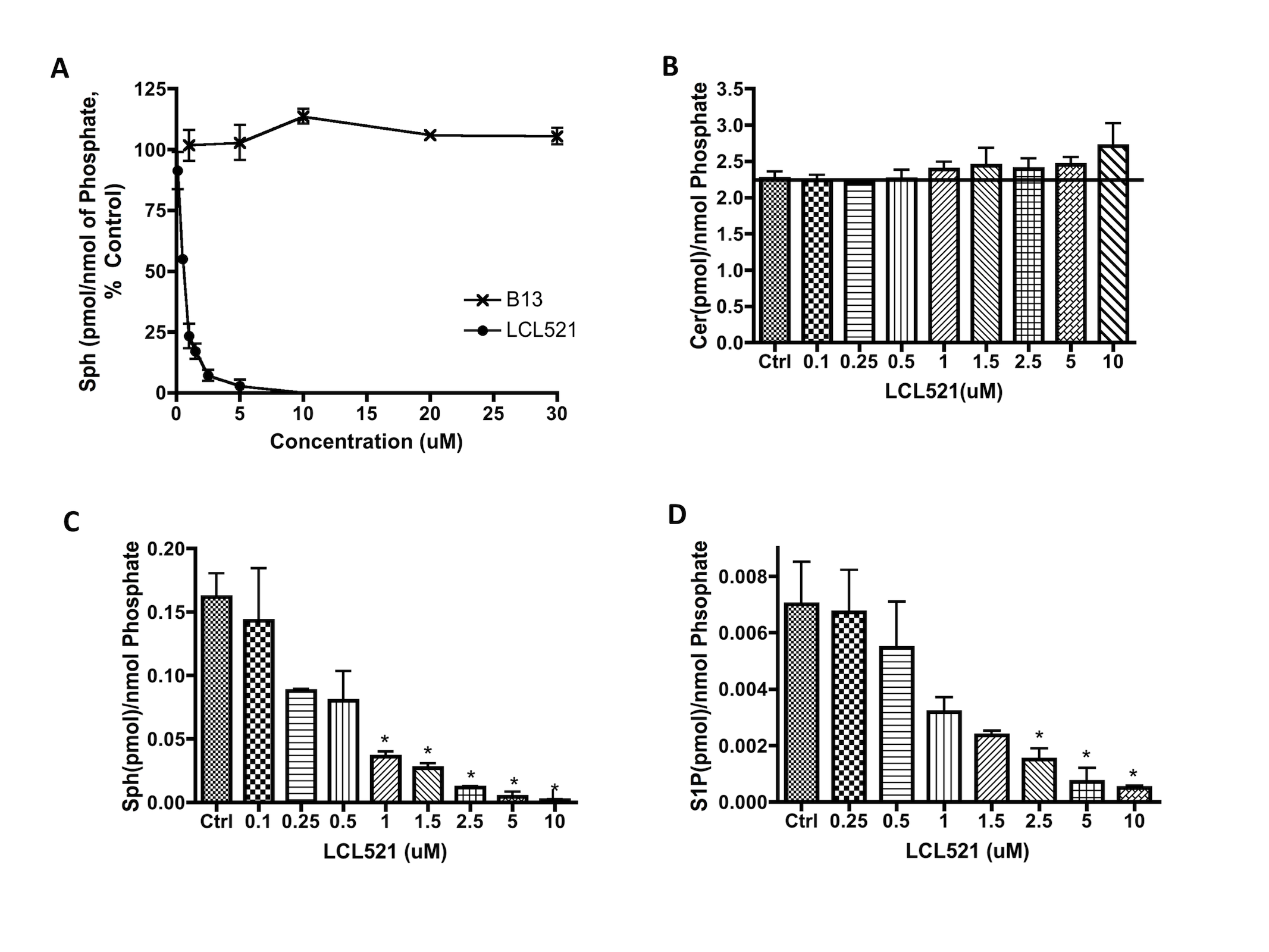

Supplement: S1 Fig — (A) MCF7 cells were treated with vehicle, or with 0.1, 0.25, 0.5, 1, 2.5, 5 and 10μM LCL521 for 1h, or with 1, 5, 10, 20, 30 μM B13 for 1h. Sph were then extracted and quantified by LC-MS/MS. (n = 2, two times experiments); (B). LCL521’s dose response on endogenous Cer. (1h, n = 2 two times experiments); (C). LCL521’s dose response on endogenous Sph. (1h, n = 2, two times experiments, * p<0.05, vs Ctrl); (D). LCL521’s dose response on endogenous S1P. (1h, n = 2, two times experiments, * p<0.05, vs Ctrl). (TIFF) [file pone.0177805.s001.tiff]

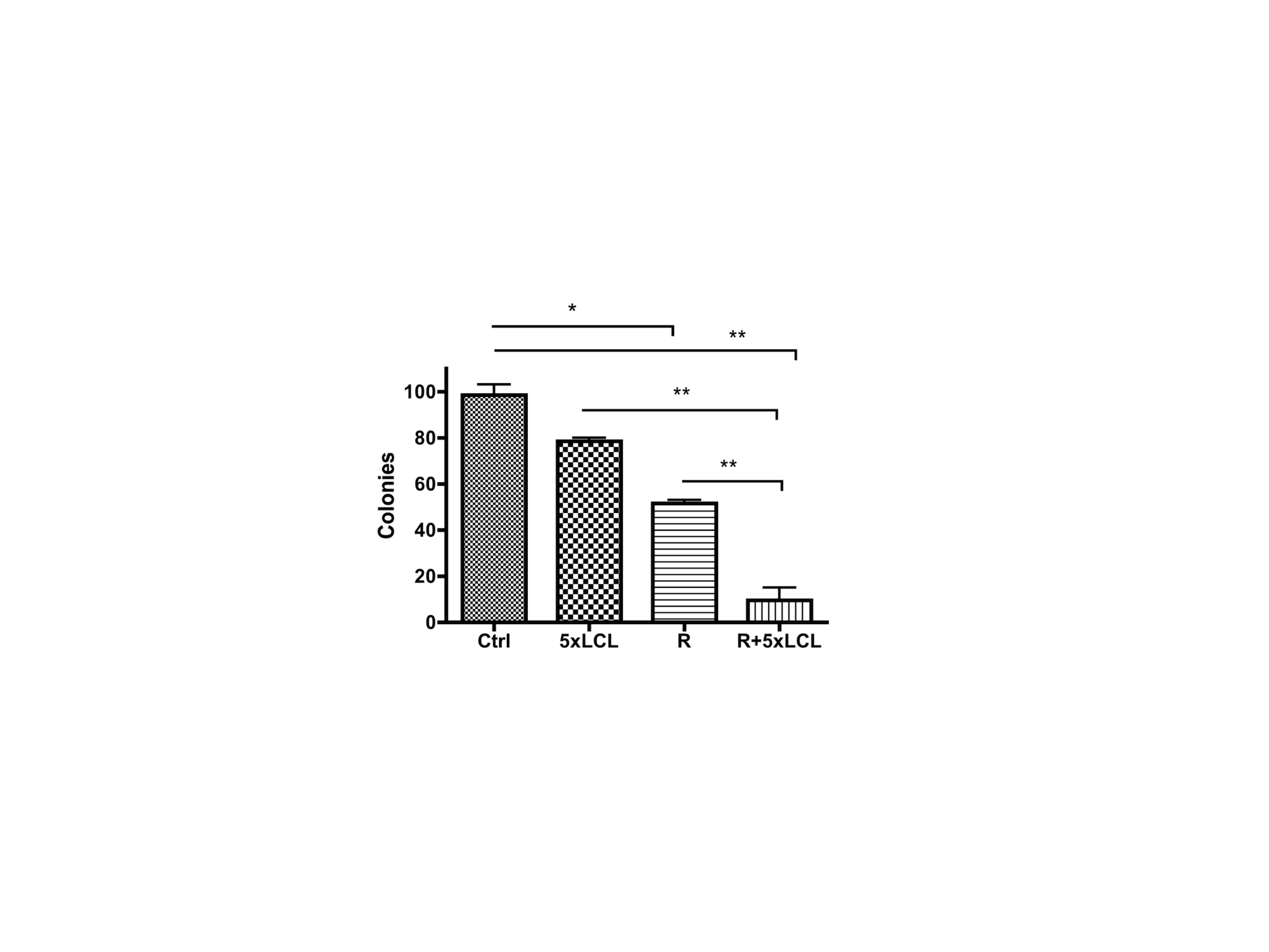

Supplement: S2 Fig — MCF7 cells were irradiated with 0 or 2Gy using 137 Cesium irradiator. 1h after IR, each replicated treatment were further treated with vehicle or 5x1μM LCL521. For the 5-time treatment, media were replaced every 24h with fresh media that contained either vehicle or 1uM LCL521. After that, cells were cultured for 4 weeks and then stained with crystal violet (1g/500ml formalin). (n = 2, two times experiments, * p<0.01, ** p<0.001). (TIFF) [file pone.0177805.s002.tiff]

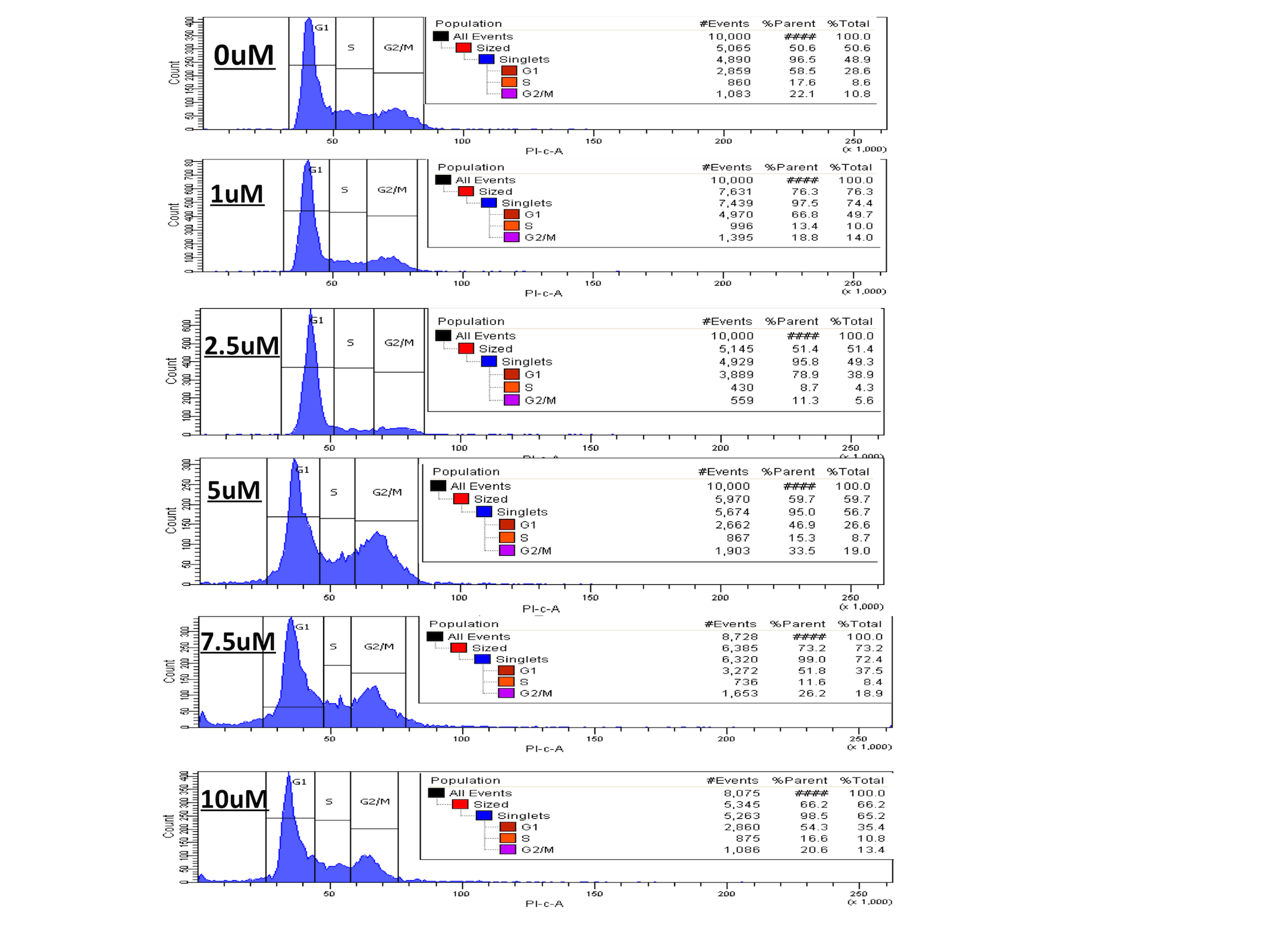

Supplement: S3 Fig — Cells were treated with vehicle or 1, 2.5, 5, 7.5 and 10μM LCL521 for 24h. Cells were then fixed with 70% ethanol overnight before adding 500μL RNase and PI solution. Samples were kept in the dark for another 45min before the FACS analysis. Representative flow cytometric analyses are shown. (TIFF) [file pone.0177805.s003.tiff]
